# Obstetrical Transactions

**Published:** 1872-11

**Authors:** 


### OBSTETRICAL TRANSACTIONS.

1. *TRANSACTIONS OF THE OBSTETRICAL SOCIETY OF LONDON. Vol. XII. For the year* 1871. *8vo. pp.* 325. *London: Longmans, Green & Co.*, 1872.

It is impossible, in the space allowed for a review, to give a full account of the numerous papers contained in these two volumes. We shall, therefore, restrict ourselves to the selection of the more important.

The London Society has an "ordinary" fellowship of nearly 600, an income of some $3,500, and holds ten sessions in the year. It is gradually accumulating a museum, library, and building fund, and has a world-wide reputation for activity and energy. The volume of its transactions before us is much less in size than some that have preceded it, and opens with the Annual Meeting in January, at which Dr. Graily Hewitt, the retiring president, delivered the annual address, and Dr. J. Braxton Hicks was chosen his successor.

Dr. Hewitt's address congratulates the Society upon its prosperity; contains obituary notices of several members deceased during the year; and reviews the work of the Society.

*Spontaneous Rupture of an Ovarian Cyst; reported with specimen.* By Dr. Protheroe Smith.—Woman, single, aged 48, bowels obstinately constipated; fibroid tumor of uterus, and ovarian cyst diagnosed. Patient seized with severe abdominal pains, followed by vomiting, collapse and death. . . Flaccid ovarian cyst of right side, ruptured at a point covered by a globular fibroid tumor the size of a small orange, rising from left angle of fundus uteri. Cyst and pelvic viscera bound down by adhesions, the result of peritonitis. Dr. Smith exhibited specimen in evidence of the necessity of performing ovariotomy early under similar circumstances.

Dr. Barnes observed that there was another mode of dealing with very early ovarian dropsy, from which good might be expected, and reported a case in which he had drawn off sixteen ounces of fluid, verified as ovarian, by means of the Aspirateur of Dieulafoy, the trocar being introduced by the vagina. Improvement followed, and the uterus retracted to its natural position. In three weeks, the bladder distress, which existed prior to the operation, returned, and it was found that the uterus was again pushed forward by the tumor. It was tapped a second time, and six ounces of fluid came away; six drachms of tincture of iodine were thrown into the cyst. The patient was again better; no marked local symptoms followed. Some weeks later, a firm mass was felt in the place of the original swelling; and as the uterus was more forward than natural, it was resolved to puncture again, in the hope that, by letting off any little exudation in it, complete shrivelling of the cyst, and a cure might be effected. But soon afterward, symptoms of irritative fever set in, attended latterly by jaundice, under which the patient sank after some weeks. Notwithstanding this issue, he thought the method deserved further clinical investigation.

Dr. Phillips referred to an American physician who advocated, and Dr. Wiltshire remarked that he believed had performed successfully, the operation of ovariotomy through the posterior wall of the vagina. . . . [This physician was Professor T. Gaillard Thomas, of the College of Physicians and Surgeons, New York, and the operation was performed February 6, 1870, terminating in entire success. See this Journal for April, 1870, page 387.]

*Irritable Bladder in the Latter Months of Pregnancy.*—Dr. W. S. Playfair refers to this sometimes distressing condition, and says: I believe that this form of irritable bladder is mainly due to a distinct mechanical cause, viz., an oblique or transverse position of the fœtus in utero, and that it is easily and effectually cured by the simple expedient of altering the position of the child by means of external pressure on the uterus. He reports three cases in illustration, in one of which he several times altered the position of the fœtus to the great relief of the patient, the change not being permanent, as it appears to have been in the others, who were all equally benefited for the time, by the altered position of the fœtus.

Dr. Barnes proposed retaining the fœtus *in situ* by mechanical means. Dr. Hewitt was at a loss to account for the irritation by the pressure of the fœtus on the bladder, because the head in the ordinary position would press quite as much as a shoulder, if not more, and that against the most irritable portion of the bladder, namely, the lower portion. He would suggest another explanation—the disturbance to the form of the bladder by the altered form of the uterus. It is well known that the vertical diameter of the bladder is increased, and its antero-posterior decreased, in the latter months of pregnancy; may not this arrangement be interfered with so as to produce the symptoms complained of?

[As a matter of interest in this connection, we will state that one case of this form of irritability that came under our care, aborted at seven months, of an anencephalous fœtus. The lady was the mother of several children, and never had suffered to the same degree before, even upon the last days of full gestation.]

*Remarks on Tables of Mortaliy aftter Obstetric Operations.* By Drs. J. Braxton Hicks and J. J. Phillips.—This paper, covering thirty pages, being itself condensed, the facts only can be stated, and deductions which were drawn from them. 1. *Craniotomy.* Smellie reports four fatal out of forty-four. Ramsbotham gives seven fatal cases. Many of the conditions out of which it was endeavored to rescue the patient were severe enough in themselves to be fatal, or at least seriously to endanger her life. It will be observed that, out of the seven cases o Ramsbotham, rupture of the uterus occurred in two, and rup-f ture of the vagina in two, and that in each of these cases the fatal accident had happened previous to any attempt at delivery. In two other cases, death resulted from sloughing, caused, as is evident from the history and the already decomposing fœtus, by the long continuance of unassisted labor. The remaining fatal case must be considered as altogether exceptional. Dr. Collins reports fifteen fatal cases. When we analyze these fifteen cases of death after the employment of craniotomy, we are at a loss to find more than two whose fatal termination can with any show of probability be traced to the operation. In most of the cases, it will be admitted by all that death arose from the too long postponement of craniotomy. Dr. Robert Lee reports sixty-seven cases, eleven of them fatal. The remarks here have the same bearing as in the former instances, and so on to the end of the paper, including the results of the use of the forceps, and the employment of version; showing the unreliability of statistical tables in establishing the mortality to be anticipated from these operations, from the fact that the complications of delay, rupture of uterus, succession of instrumental expedients, etc., increase the mortality in the various cases, which cannot be properly associated under one head, either as forceps cases, craniotomy, or version. The whole paper is one well calculated to show the fallaciousness of the tables upon which the statistics of mortality, after the obstetric operations referred to have been based. The remarks of Dr. Phillips at the close of the discussion which followed the reading of the paper, will make a suitable explanatory ending to the imperfect sketch here given. He said that he was pleased to find that the general experience of the Society declared in favor of the belief expressed in the paper, that the mortality after obstetric operations in a large proportion of cases was wrongly attributed to the operations themselves. Probably many accoucheurs were influenced in delaying instrumental assistance by the high rate of mortality assigned to craniotomy, or the use of the forceps. The practical lessons were easily deduced, but with these, Dr. Hicks and himself were not on that occasion specially concerned; their chief object was to prove the unscientific manner in which the ordinary tables of mortality had been compiled, and to call attention to the wrong conclusions which had been drawn from them.

Extensive researches into the history of the Cæsarean operation, as performed in this country, have led the writer of this to a similar conclusion, the mortality being due less to the gravity of the operation than to all the accompanying circumstances of delay, fruitless attempts at instrumental delivery, etc.

*A Case in which the entire Ovum was Expelled at the Seventh Month, and the Child rescued alive.* By Dr. John Brunton.—The woman, aged 32, had previously given birth to four children, and on the occasion reported, which was in December, aborted of twins, the second being inclosed in the membranes for a period of time calculated at about fifteen minutes, before they were opened, when the child cried, and appeared in a better condition than the first; both died the following day, the second living much the longer of the two.

This report has a bearing upon the subject of intra-uterine fœtal independence, and especially upon the possibility of saving the child in placenta prævia after practicing the method of Simpson in separating the placenta from the arrest of hemorrhage. Dr. Brunton states that in a case under his care the child was saved after the placenta had been removed and upon the bed for ten minutes.

*The Vomiting of Pregnancy—its Causes and Treatment.* By Graily Hewitt, M.D.—The purport of this article of nineteen pages is as follows: He says, having had occasion to treat cases of sickness in young unmarried women suffering from flexion, it has been observed by me that when those patients marry and become pregnant, the sickness observed is liable to be unusually severe and troublesome. Regarding the cases which have come to me in the course of consultation practice, and where the symptoms of sickness have been still more troublesome, the same fact holds good, the connection between the two things has been observed to exist. He relates a typical case in which anteflexion was the cause of trouble, which was relieved by placing the woman upon her back in bed, and confining her to this position, in order to allow the expanding uterus a better chance of escaping from the pelvis, and that the bowels should be kept regularly open. He says in explanation: the tissues of the uterus, including the nerve ramifications in it, are compressed at the seat of flexion, and this is the cause of the sickness. He recommended the use of pessaries as a means of cure.

Dr. Barnes observed, there had been many theories advanced as to the cause of vomiting in pregnancy. Displacement of the uterus, as retroflexion and anteflexion, was an old theory. It was mentioned by Moreau; and Mayer and Ulrich give cases in which anteflexion was the cause. He was in a position to state, from many precise observations, that flexions of the gravid uterus were often present without any unusual degree of vomiting, and that the most obstinate vomiting occurred where there was no flexion. He believed in the theory of the stretching of the uterine fibre, as set forth by Bretonneau of Tours, and brought forth in evidence the facts of vomiting resulting from intra-uterine polypus; the injection of water to induce labor; the rapid accumulation of an excess of amnii; and the existence of twin pregnancy. His theory was, that growth that kept pace with that of the contents of the uterus did not cause vomiting; but it was caused whenever the fibre was stretched rapidly, the distending contents out-running the accommodating growth of the uterus.

In a long-continued criticism which followed, the views of Dr. Barnes were generally concurred in, and the employment of pessaries for mechanical support condemned as unsafe.

*Case of Cœsarean Section.* By Henry Gibbons, M.R.C.S. The subject was three feet ten inches high, twenty-two years old, and in labor five hours. The steps of the operation were all favorable, the incision having been made prior to the rupture of the membranes, and everything bid fair for the recovery of the patient. Chloroform was employed during the operation, and in about an hour a full dose of opium was administered. Uncontrollable vomiting commenced soon after, and continued with scarcely any intermission until she sank, the morning of the 15th January, or about forty hours after the operation. The examination of the body showed neither extravasation into the peritoneum, nor any peritonitis. The child lived nine days.

We would ask whether death resulted as a secondary effect of chloroform, or immediately, from the operation? In the reviewer's table of sixty American cases, there is no parallel instance of death. The vomiting followed too quickly the dose of opium to have resulted from it.

*Case of a Child Born at twenty-two weeks and two days, living for eighteen days after Birth.*—The mother was thirty-eight years old, had had nine children and three miscarriages, and the date of the conception noted appears to be well established. The fœtus was a male, and the testicles had not descended into the scrotum. Dr. C. H. F. Routh, who reports the case, mentions that of one born living at four months; another reported by Maissonneuve as respiring for six hours at four and a half months; and a third by Mr. Smyth, twelve hours, at five months. We may add another in our own experience, similar to the last, born of a woman far advanced in phthisis.

*On Tetanus after Abortion.* By Alfred Wiltshire, M.D.—This is the title of an article covering six pages, founded upon two cases which are reported, in both of which there existed great mental depression, one arising from the fact of the conception being adulterous, and the other from cruel desertion by her husband. This is a subject of considerable interest, although of little practical value, as the disease is of great rarity in temperate climates. In hot countries where traumatic tetanus is not uncommon, puerperal tetanus is also frequently met with.

Dr. Playfair remarked that he had seen many cases in the Lying-in Hospital at Calcutta, even when there was nothing otherwise abnormal about the patient which would favor its occurrence.

*On Pelvic Hœmatocele, with special Reference to its Diagnosis and Treatment.* By Dr. Alfred Meadows.—This article and its discussion constitute a valuable treatise upon this form of disease. There appears to be a considerable difference of opinion as to the frequency of the malady, its diagnostic features, its extremes of gravity, and the necessity or propriety of evacuating the blood through the vagina or rectum. It is impossible to make a satisfactory summary of the mass of evidence, suffice it to say, that it leaves the impression that a careful tactile and symptomatic diagnosis would reveal the existence of the disease in mild, as well as its graver forms; and experience is in favor of evacuating the fluid where it is large in quantity, not very recently effused, and is producing by its presence severe constitutional and local disturbance. There is abundant evidence to show that the peritoneal cavity is much more tolerant of the presence of blood than of any other extraneous fluid, and rarely exhibits the signs of peritonitis as a consequence of its effusion. In retro-uterine hæmatocele the blood in time becomes encysted in some cases, and subsequently undergoes a change by which it is rendered putrescent, and a source of constitutional irritation, when it is well to liberate it by puncture.

We have seen the peritoneal cavity so largely filled with blood, that its loss from the circulation reduced the patient apparently to a moribund condition, and still there was no peritonitis resulting from its presence, or symptoms requiring its evacuation; the lady recovered her health perfectly, and several years subsequently gave birth to a healthy infant.

Dr. Robert Barnes remarks: My own observations lead me to the opinion that the true indications for puncture are constitutional evidence of blood-poisoning from alteration in the blood-mass of the tumor; or distressing pressure upon the bladder and other pelvic viscera; and that in those cases where there is no evidence of such conditions the prospect of recovery is in no sensible degree enhanced by puncture, and that the practice, therefore, is to be shunned simply because too much diligence is bad.

Dr. Protheroe Smith recommends that the fluid, when evacuated, should be drawn off by the aspirator, and says of its use in one case: By it every particle of matter and gas was sucked out so completely that perfect collapse of the sac took place, there was no reformation of pus, and the patient left the hospital apparently perfectly well.

*On a Case of Sudden Death Seventeen Days after Delivery.* By Dr. W. S. Playfair.—The subject was a healthy lady, thirty years of age, primipara, and delivered after a short and easy labor. Convalescence progressed favorably until the tenth day, when fever, embarrassed respiration, and physical signs of pleurisy resulting from exposure to a draught, appeared. On the fifteenth day was apparently perfectly restored. On the seventeenth day ate a hearty breakfast and was anxious to get up; about noon, suddenly, whilst sitting up in bed, was attacked with cardiac pain, great dyspnœa, became livid in countenance, and was dead in about five minutes. No autopsy. Dr. Fairplay remarks in explanation of the cause of death: The slight pleuritic attack had produced an increased hyperinosis of the blood, over that already existing from the alteration due to the puerperal state. Hence the coagulation, which probably might not have occurred had it not been for the apparently trivial and temporary pleuritic mischief, which, under ordinary circumstances, would have given rise to no anxiety.

*On the Diagnosis of the least known Varieties of Uterine Inflammation.* By Dr. E. J. Tilt.—This article, with the ensuing discussion, cannot from its nature be reproduced in abstract form.

It is a paper of much value and well worthy of examination; the author condemns the effort made by Dr. Routh, to distinguish fundal endometritis from internal metritis.

*On the Contractions of the Uterus throughout Pregnancy; their Physiological Effects, and their Value in the Diagnosis of Pregnancy.* By Dr. J. Braxton Hicks.—In this well-written article the author gives the results of his examinations of the gravid and otherwise enlarged uterus, through the abdominal walls, during a period of eight years, and establishes the fact of the continued recurrence of contraction and relaxation in the muscular fibres of this organ during the last six months of pregnancy particularly, but in all cases, where it is distended to a large size by any internal growth.

Dr. Hicks remarks: If the uterus be examined without friction, or any pressure beyond that necessary for full contact of the hand, continuously, over a period of from five to twenty minutes, it will be noticed to become firm at first, and more or less flaccid, if it be firm at first. It is seldom that so long an interval occurs as that of twenty minutes; most frequently it occurs every five or ten minutes, sometimes even twice in five minutes. However, in some cases I have found only one contraction in thirty minutes. . . The constancy with which these contractions of the uterus have already occurred to me, leaves no doubt on my mind but that it is a natural condition of pregnancy irrespective of external irritation. In a general way the pregnant woman is not conscious of these contractions. But occasionally it happens that the uterus is more than usually sensitive, and that the contractions are accompanied by pain; and then on examination it is found that each pain she complains of is coincident with a contraction.

Again, when the uterus has been excited by any cause, and these contractions are more than usually powerful, the woman is conscious of their presence, and by watching these we shall convince ourselves that the contractions which were before unnoticed by her, are really the same as the so-called *pains* of premature expulsion of the fœtus and also of true labor.

These remarks of Dr. Hicks remind us of a case in which this condition existed almost to the extent of expulsion. The patient was the wife of a physician, and was pregnant the second time, having miscarried with her first child, which lived but three days. When we were called to see her in July, 1864, we found that she had been suffering with recurrent painful contractions of the uterus for some days, which her husband had not succeeded in controlling. She had had no discharge per vaginam, although her symptoms closely resembled those of actual labor. When the hand was placed upon the abdomen, the contraction and relaxation were very distinctly noticeable in the walls of the uterus at each recurrence; and the expression of the woman alternately indicated pain and relief. Under the use of morphia the action of the uterus gradually diminished, until at the end of ten days she was entirely relieved. She was delivered of a living child in October. The painful contractions in this case continued altogether about three weeks.

Dr. Hicks proposes to make a practical use of this habit of the uterus, to distinguish it when enlarged from non-uterine tumors, and to detect the presence of abnormal intra-uterine growths and fluid collections by the comparative differences under palpation of the contracted and relaxed pregnant uterus; and the state under similar conditions, when distended by solid tumors, hydatids, and fluid accumulations.

*Abstract of a Memoir on Osteomalacia, by Dr. Gaetano Casati, of Milan*, by Dr Robert Barnes.—Although this disease is almost unknown in the United States, it will be of interest to a portion of this paper as explaining the reasons for the prevalence of the malady in some European localities, and per contra, its non-existence in any special district here.

Dr. Casati gives the experience of eight years in the Royal School of Midwifery at Milan, during which period 7719 women were delivered, of whom sixty-two presented evidences of clearly-marked osteomalacia:

Almost all the women came from that part of the province of Milan known as the Valley of Olona. All the women came from wretched villages where food was scarce, consisting for the most part of maize or rice, often musty; they worked at washing or weaving; were badly lodged, their rooms being ill-ventilated, out of repair, the glass of the windows generally substituted by paper; the houses built upon clay, and damp.

Added to these unhappy conditions, Casati thinks the character of the water and the vegetables with which the people eke out their scanty diet is largely concerned.

Only one of the sixty-two women was a primipara, and excepting five, all had had three children or more; forty-one presented various degrees of pelvic deformity. One-half of the women were delivered normally, and of the remainder, but five required the reduction of the fœtal head, and two, the operation of Cæsarean section; forty-two children survived.

It will thus be seen, that until we have wretched, ill-fed, crowded peasantry in the United States, living in damp localities upon the ground, we are not likely to meet with osteomalacia, except in very rare instances; and that it is only in the minority of cases that destruction of the fœtus or the Cæsarean operation is required.

*A Case of Extra-uterine Gestation, with Remarks on the Treatment of that Condition*, by Dr. ALFRED MEADOWS.—This article presents the simple record of a case of abdominal pregnancy, in which a young woman died from rupture of the cyst and consequent internal hemorrhage, without any attempt having been made to prevent the occurrence of the accident. Dr. Meadows reflects somewhat upon himself for not having anticipated the event by the operation of gastrotomy, and proposes in such an operation to tie the fœtal cord and leave the placenta, instead of running the risks of hemorrhage by its detachment. The reading of this paper provoked a lengthy discussion upon the subjects of the diagnosis of extra-uterine pregnancy, its dangers, spontaneous cures, and the propriety and methods of surgical interference: from which it may be inferred that they thought it proper to open the abdomen in cases where rupture had already occurred, and would also recommend the anticipatory operation if the diagnosis was not so uncertain, and patients did not sometimes recover by calcification of the fœtus, or its discharge by abscess and ulceration into the vagina, rectum, or through the abdominal walls. The great obstacles to the early operation as expressed by Dr. Hewitt and Playfair, are, the difficulties of satisfactory and certain diagnosis; the fact that cases rarely present themselves until too late for gastrotomy as an anticipatory measure; the difficulties to be met with in the form of extensive adhesions; and the dangers of uncontrollable hemorrhage. Dr. Meadows took the bolder plan, and recommended the abdominal section as soon as the diagnosis could be clearly established, regarding it as less dangerous in the average, than waiting the course of nature.

*Successful Case of Ovariotomy during Pregnancy, with Remarks on the Treatment of Ovarian Tumors complicating Pregnancy*, by EUGENE GODDARD, L.R.C.P.; operation by Spencer Wells.—The subject was a lady 29 years old, pregnant for the sixth time, and about two months advanced at the date of the operation. Incision five inches long; eleven and a half pints of fluid removed; weight of cyst and solid matter three and a quarter pounds. Total about fifteen pounds. Anæsthetic used, chloro-methyl. Recovery from operation rapid and complete; patient went to full maturity; labor natural; able to nurse her infant, and in as good health as after previous confinements.

In his defense of the operation during gestation, Dr. Goddard remarks:

Ovariotomy, now an accepted operation, has had to fight its way through more opposition and bitter prejudice than perhaps any other. Sufficient facts have, however, been accumulated to establish it now as one of the triumphs of modern surgery. But it seems that a new development is open to it. The peritoneum, it is settled, may be invaded under certain careful precautions, perhaps the most important being that no ovarian fluid be allowed to escape into its cavity; or if it do, that it be carefully removed. May we not now say the peritoneum may be safely invaded under like conditions during pregnancy? Or if it be objected that the cases are still too few to warrant such a conclusion, may we not say that the tendency of the cases on record, is to prove that ovariotomy has been thus far so good an alternative that it is only necessary it should be followed up to become established as in many cases the *best* alternative in this complication?

The president remarked that in the case of a compound or solid cyst, if relief must be given, then the choice lay between induction of labor and ovariotomy. The cases of Spencer Wells showed, so far as they went, that the results of the latter had been satisfactory, and no doubt in his hands the question would receive an intelligent solution.

*Fibrous Enlargement of the Uterus successfully treated by Ergot*, by Dr. JOHN BRUNTON.—Subject, a single woman of 47; fair complexion, and in full flesh. Uterus when first examined reached midway between pubes and umbilicus. Menses excessive, and periods about every two weeks. Treated at first by iodine to abdomen, and bromide of potassium internally, but with no benefit; then with digitalis, morphia, cannabis indica, and finally ergot. The hemorrhage becoming alarming, and the uterus reaching as high as the umbilicus, the ergot was administered in large doses, as much as from one to two ounces being given at each period, with controlling effect. The uterus began to diminish in size under this treatment, and in six or seven months could not be detected above the pelvis, and there was little or no increase in its size, examined by the rectum. She is reported to have taken from forty to fifty ounces of ergot in six months, and to have had morphia injected hypodermically very often, sometimes daily. At last report, the catamenia were still frequent and excessive, but under control by ergot and morphia; no enlargement of the uterus could be detected.

2. *TRANSACTIONS OF THE EDINBURGH OBSTETRICAL SOCIETY. Session* 1868-69. *8vo. pp.* 207. *Vol. I. Edinburgh: Maclachlan & Stewart*, 1870.

The Edinburgh Obstetrical Society was instituted in 1840, but did not publish its transactions in book form until 1870, at which time there were 109 "Ordinary," and 105 Corresponding Fellows upon its roll. The work opens with a monograph of fourteen pages, entitled:

*On the Construction of the Cephalotribe*, by Dr. J. MATTHEWS DUNCAN, M. D.—This article is intended to point out the errors in the construction of the short handled, or Edinburgh instrument (Sir James Y. Simpson's); and instituted a comparison between its efficiency and that of the Paris instrument (a modification of Baudelocque's). Dr. Duncan made a series of experiments upon the relative powers of the two instruments, and condemns the short handled variety as deficient in leverage; not shutting the blades closely enough before the handles are brought in contact; and having too much spring in the former, without there being any compensation of the handles, which should pass over each other. He recommends a slight modification of the Paris instrument, giving it a strong button-pivot, thin but broad handles, and a free passage of the latter over each other, so as to allow the screw to be turned until the handle ends are in close contact, thus reducing the weight from 4 lbs. 1 oz. to 3 lbs. 5 oz., without diminishing the necessary strength of the cephalotribe.

The reading of this paper occasioned a considerable discussion, parties admitting the value of many of the views advanced by Dr. Duncan, but at the same time claiming an efficiency in the Edinburgh instrument far beyond what he had apportioned it; Dr. Keiller stating that he had repeatedly used the condemned cephalotribe, and had had no great difficulty in crushing the fœtal head with it. The general impression appears to have been, that the instrument of Dr. Duncan was unnecessarily cumbersome.

*Note of a Case of Puerperal Mania*, by Dr. MENZIES.—This is chiefly valuable as an instance of the effect of bromide of potassium, the patient having taken four ounces during six weeks, with an intermission of eight days. The notes of the case from day to day do not show any very satisfactory effects from the remedy. Dr. Wilson thought that the recovery of the patient had not been more rapid than many cases of mania treated without the bromide.

Dr. Ritchie mentioned a case in which the bromide failed, the patient became much reduced, would take no food, and ultimately died. He held the opinion that puerperal mania was in most instances the result of nervous exhaustion and required a stimulating treatment.

*Birth of a Child weighing seventeen Pounds*, by PETER BROTHERSTON, F.R.C.S.E.—The mother was five feet high, aged 40, and in labor with her seventh child. The head came away normally, but the body could not be delivered by manual assistance, and the child perished before it was finally removed by the blunt-hook. Length of fœtus, 22½ inches; occipito-frontal circumference, 15 inches; circumference of shoulders, 20; chest, 15½; abdomen, 17; funis to head, 16; to sole of foot, 15½; length of arm, 10 inches. The child was perfectly formed in every respect. The father was 44, six feet high, and weighed 182 pounds. So much force was required in delivering the child that its right humerus gave way near the neck under the pressure of the blunt-hook.

*How long may a Dead Fœtus remain in the Uterus?*—This question is propounded by Dr. James Young, who relates the case of a woman æt. 36, mother of four children, who, becoming insane, was sent to an asylum, where she in a measure recovered, returned home, conceived, was recommitted for three months, after which she became well, and so continued. She became pregnant during six days when at home in January, and was not again home until April, felt the motions of the fœtus until the end of June, after which there was no more motion or increase of size, and breasts became small and flaccid. Fœtus expelled in September, putrid, and inclosed in the membranes; measured when extended, ten inches. Dr. Young computes the age of the fœtus at its death at five calendar months and ten days, and that the mother continued to carry it two months and ten days longer, without any detriment to her health.

Mr. Pridie reported a case in which he made the calculation that more than three months had expired between the death of the fœtus and its expulsion. The fœtus died from the effects of fear, excitement, and over-exertion on the part of the mother in escaping from a party of cavalry on drill.

*Transverse Construction of the Inferior Straight; Caries of Ischium, with Sequestrum projection in Vagina; Cephalotripsy, and Recovery,* by Dr. James Grenser, of Dresden.—Woman unmarried, æt. 20; four feet and three inches in height. Tubera ischii closely approximated, scarcely admitting two fingers; one inch above the vulva a splinter of bone, about an inch and a quarter long, was found projecting into the vagina, and arising from the ascending ramus of the right ischium. This bone was removed without hemorrhage; labor continued sixty-eight hours before full dilation of os uteri was effected. Forceps then applied, which occasioned considerable hemorrhage, supposed to come from the carious ischium; head drawn down, perforated, crushed with cephalotribe of Busche, and child delivered. Woman left the hospital on the ninth day. This carious condition of the pelvis had existed from the age of seven, at which time of life she had vaginitis, purulent discharge, and subsequently escape of fragments of dead bone. For nine years prior to her labor, the discharge had been slight. The inferior straight measured 3 inches 4 lines antero-posteriorly, and 2 inches 2 lines between the tubera ischii.

*Caoutchouc Dilator.*—Dr. Keiller remarked:

The mode of inducing premature labor by means of caoutchouc bags was first brought before the Society (as reference to its proceedings will prove), and several Fellows now present had been made practically conversant with this plan before it was adopted by the gentleman whose name it now generally bears.

Sir James Simpson and other members replied, that if any name ought to be attached to this special mode of practice, it was unquestionably that of Dr. Keiller, and not of Dr. Barnes. It was only fair to Dr. Keiller and to the Society that this should be known and acknowledged.

*Tetrachloride of Carbon.*—Two cases of labor reported by Dr. Inglis, in which this anæsthetic was used. First patient took it undiluted, resulting in violent uterine action in first stage of labor, and syncope under its full impression. In second case (one of uterine inertia), given with an equal portion of chloroform, resulted in bringing an effective expulsive action at once.

Sir James Simpson found that it depressed the pulse, but did not find it produced syncope; had used nitrate of amyl, but it did not succeed in labor, although a pain-reliever in dysmenorrhœa, cancer, headache, etc.

*Spontaneous Healing of Harelip in Utero.*—Case reported by Dr. Cuthbert.—Scar well marked on both sides of lip, soft palate fissured. Child being at age of three months when reported.

*Complete Separation of the Placenta before Labor without external loss of Blood.*—Dr. James Cappie reports two cases where apparently the whole placenta had become suddenly detached from the inner surface of the uterus. The first accident happened as the woman was getting out of bed in the morning, in the seventh month of her pregnancy, having been aroused by severe abdominal pain. She was delivered as soon as practicable with the forceps. The placenta was remarkably soft, and the child affected with general anasarca. The mother was pale and delicate, but had no albuminuria. The second patient was in excellent health, and like the other in her fifth pregnancy, but advanced about seven and a half months, was suddenly seized, without assignable cause, with abdominal pain followed by giddiness and fainting, and upon recovering consciousness experienced a sensation of increased distention. Os uteri found closed; no evidence of labor; forcibly introduced the finger and commenced dilatation, but without separating the membranes to any extent; labor excited, advanced rapidly; child delivered in a flaccid, anæmic state in less than two hours. Placenta presented a tear in its centre, but was otherwise healthy. Both women recovered. The reasons given for believing in the entire separation of the placentæ were, that in both cases they were extracted before any clots were discharged; were found low in the vagina, and the membranes inverted by the gush of discharge, carrying the placentæ in advance. The external character of the uterus under palpation, in both instances, appears to have given evidence in favor of entire separation, a very rare condition as compared with the number of cases which are only partial.

*Cephalotripsy after Turning*, by Dr. KELLAR.—In this case the woman was in her second labor, the first having been terminated by instruments after several days' suffering. Dr. Pridie was engaged to attend her with the second, and called in the aid of Dr. K. Turning was resorted to in preference to craniotomy, in the hope of possibly saving the child, but when the body was extracted the impossibility of delivering the head entire was discovered; it was then perforated, the body slowly drawn upon, and pressure made upon the head from above the pubis, when at the maximum of effort the body separated at the neck. Forceps, crotchet, craniotomy forceps, and cranioclast were tried in succession, but failed. The cephalotribe of Simpson was then obtained, and the head crushed and removed. The woman made a good recovery. Dr. Keiller advocates the "Edinburgh Cephalotribe," as that of Professor Simpson is generally called, in preference to the longer and heavier modifications of Baudelocque.

*On the Scarification of the Gums of Infants in Dentition*, by Dr. Cairns.—This article is an effort to prove that the operation does not relieve pain, remove tension, prevent or check convulsions and diarrhœa, and is a preventive of the subsequent ready protrusion of the tooth by the formation of a dense cicatrix. In the discussion which ensued the current of opinion was decidedly against him, the only concession made being that the operation was performed oftener than there was any necessity for.

*On the Combined External and Internal Method of Version*, with cases, by Dr. Alexander Milne.—This article is written in advocacy of the method of delivery by the feet, instead of the use of the forceps, Dr. M. believing from his experience of forty cases of version by the old method, that under chloroform, the operation is attendant with very little danger. He recommends in cases where the os is rigid, or but slightly dilated, and especially where the membranes are intact, that the bi-manual method of Braxton Hicks should be employed. He reports four cases in which he used the combined method. The first, Presentation of head and funis—version readily accomplished before rupture of membranes. Second, Moderately contracted pelvis, woman delivered once by forceps, and twice by turning; os small and rigid, version as before, but more difficult, as the amniotic fluid was believed scanty, and the abdominal walls were greater in thickness. Third, Placenta prævia with profuse flooding—version accomplished mainly by external pressure, woman and child both saved. Fourth, Convulsion, no labor-pains, used forcible dilatation with fingers, version before rupture of membranes, both saved.

*On the Use of the Chlorate of Potash in Certain Cases of Pregnancy*, by Dr. Cuthbert.—Case reported where five abortions had occurred in six years after marriage, in all of which the placentæ was diseased except one, in which the labor was induced by a fall at the sixth month. In a sixth pregnancy was kept much of the time in a recumbent position, and under the effects of the chlorate of potash: gave birth to a healthy child.

In the discussion which ensued, much credit was given to the chlorate, in similar cases. Sir James Y. Simpson had used it in many instances, and recommended it in doses of ten to twenty grains three times a day, combined with rest.

*Consecutive Malformation.*—As an instance of this, Dr. Menzies reports three successive births of children affected with spina bifida, the offspring of the same mother. The reviewer has a patient whose first and second children are hypospadiacs, and whose only sister has a son similarly malformed. It is not hereditary in either instance.

*Ovarian Cyst ruptured by Accident, resulting in Cure*, by THOS. L. M'MILLAN, L.R.C.S.E.—The subject was single, aged forty-two, laboring under chronic bronchitis, emaciated and somewhat cyanotic, case deemed very unfavorable; was knocked down and injured by a runaway horse attached to a cart; taken up insensible, and remained unconscious for several days; upon recovering her senses, found abdominal tumor had disappeared. After being confined to bed six weeks, and suffering with a severe cough, she entirely regained her health in every particular, and was permanently cured of her dropsy.

*Two Cases of Convulsions during Dentition arrested by Scarification of the Gums*, by G. STEVENSON SMITH, L.R.C.S.E.—This is an answer to the paper by Dr. Cairns, previously quoted, taking an entirely opposite view of the value of scarification, and giving cases in evidence of its power to arrest convulsions, relieve pain, etc. His opinions were sustained by the views and experience of the several Fellows who took part in the discussion of the question. R. P. H.

*American Journal of Medical Sciences.*

